# UWB Channel Impulse Responses for Positioning in Complex Environments: A Detailed Feature Analysis †

**DOI:** 10.3390/s19245547

**Published:** 2019-12-16

**Authors:** Sebastian Kram, Maximilian Stahlke, Tobias Feigl, Jochen Seitz, Jörn Thielecke

**Affiliations:** 1 Fraunhofer IIS, Am Wolfsmantel 33, 91058 Erlangen, Germany; stahlkema64636@th-nuernberg.de (M.S.); tobias.feigl@fau.de (T.F.); jochen.seitz@iis.fraunhofer.de (J.S.); 2 Institute of Information Technology (Communication Electronics), Friedrich-Alexander University (FAU), 91058 Erlangen-Nürnberg, Germany; joern.thielecke@fau.de; 3 Georg Simon Ohm Institute of Technology, 90489 Nürnberg, Germany; 4 Programming Systems Group, Friedrich-Alexander University (FAU), 91058 Erlangen-Nürnberg, Germany

**Keywords:** Ultra-Wideband, positioning, channel modeling, machine learning, feature extraction

## Abstract

Radio signal-based positioning in environments with complex propagation paths is a challenging task for classical positioning methods. For example, in a typical industrial environment, objects such as machines and workpieces cause reflections, diffractions, and absorptions, which are not taken into account by classical lateration methods and may lead to erroneous positions. Only a few data-driven methods developed in recent years can deal with these irregularities in the propagation paths or use them as additional information for positioning. These methods exploit the channel impulse responses (CIR) that are detected by ultra-wideband radio systems for positioning. These CIRs embed the signal properties of the underlying propagation paths that represent the environment. This article describes a feature-based localization approach that exploits machine-learning to derive characteristic information of the CIR signal for positioning. The approach is complete without highly time-synchronized receiver or arrival times. Various features were investigated based on signal propagation models for complex environments. These features were then assessed qualitatively based on their spatial relationship to objects and their contribution to a more accurate position estimation. Three datasets collected in environments of varying degrees of complexity were analyzed. The evaluation of the experiments showed that a clear relationship between the features and the environment indicates that features in complex propagation environments improve positional accuracy. A quantitative assessment of the features was made based on a hierarchical classification of stratified regions within the environment. Classification accuracies of over 90% could be achieved for region sizes of about 0.1 m${}^{2}$. An application-driven evaluation was made to distinguish between different screwing processes on a car door based on CIR measures. While in a static environment, even with a single infrastructure tag, nearly error-free classification could be achieved, the accuracy of changes in the environment decreases rapidly. To adapt to changes in the environment, the models were retrained with a small amount of CIR data. This increased performance considerably. The proposed approach results in highly accurate classification, even with a reduced infrastructure of one or two tags, and is easily adaptable to new environments. In addition, the approach does not require calibration or synchronization of the positioning system or the installation of a reference system.

## 1. Introduction

Positioning in various environments has been the subject of intense research in recent years as demand for location-based services has increased, especially in the industrial sector. In particular, RF-based systems have been used for more and more applications with a great variety of cost, complexity, and performance. Typically, in many outdoor environments, a LOS (line-of-sight) link between the receiver and the transmitter can be assumed, providing ideal conditions for many RF-based systems. However, unfavorable and complex environments still pose a challenge to RF-based positioning because diffraction, reflection, and absorption are difficult to describe via reliable analytical models. Industrial surveillance via automatic identification, detection, and localization of production tasks is one of the key components of smart industry processes. Typically, an industrial environment poses a challenge to RF-based positioning: workpieces, metal furniture, machinery, and similar obstacles often obstruct the LOS between targets and infrastructure nodes. In addition, the effective use of the production space leads to dynamic changes in the environment and thus to changes in the signal propagation. For example, classical methods based on multilateration of the signal arrival times cannot handle the complexity of such radio channels and lead to erroneous position estimates. In recent years, various data-driven methods have been developed that can handle these complex signal propagation conditions, or even exploit them to improve position estimates. However, these methods use signal propagation properties in combination with multilateration techniques and thus require an elaborate time synchronization system.

Concerning the propagation of radio signals, ultra-wideband systems (UWB) have recently been found to be a low cost, reliable, and scalable technology. Due to the high bandwidth, they cause only low interference with other RF positioning and communication systems such as Bluetooth and WiFi and can be operated in parallel. Due to the signal design commonly used in UWB systems, it is possible to extract channel impulse responses (CIRs) from recorded data. These CIRs contain information about the propagation paths of the signal. Therefore, the CIRs contain a variety of information, apart from the time of arrival (ToA) or similar signal characteristic commonly used for positioning. Especially in a complex scenario such as an industrial environment, the signal propagation conditions, and thus the results of the collected CIRs, can drastically vary with the arrangement of the objects along the propagation path. The complexity of the environment is difficult to capture in an analytical model. Therefore, data-driven machine learning (ML) is used. These methods can use spatially significant irregularities in the CIR data, rather than suffering from it, to improve positional accuracy. In general, a CIR can provide a comprehensive set of information.

The main problem with the use of state-of-the-art ML positioning methods is the amount of labeled data that is necessary to train the ML methods. Thus, to train such a model for accurate positioning, a reference system must be installed, at least temporarily. Here, technology experts are needed to generate labeled data records that can be processed in the ML framework. However, these records lose their validity over time as the environment may change. Moreover, in a production environment, semantic interpretation is often more important than high-precision localization. Employees find it more intuitive to understand semantic information as geometrical information (for example, between shelf A and B rather than at the position (x = 32.5 m, y = 20.1 m). This semantic interpretation allows a simple and quick labeling process for which no time-consuming and precise labeling procedure is required, such as installing a complex reference system.

By extracting only relevant features, the information contained in CIRs is compressed using existing knowledge about the propagation of radio signals. Therefore, the resulting models are less complex and have lower computational requirements for training the models and for inference at runtime. This article explains the contribution of each feature to localization accuracy from recorded CIRs that meet different propagation conditions. This allows a qualitative assessment of the proposed positioning features.

Data from a free space propagation scenario were compared to a scenario with reflector and absorber walls (i.e., objects that have a traceable influence on the propagation channel). These datasets were used to distinguish the behavior of the CIRs in LOS and NLOS channels. In addition, data from a realistic industrial scenario were evaluated, including several disturbing objects such as metal storage racks and industrial vehicles. The recorded contained both the CIRs from multiple infrastructure tags within the environment and positions provided by an accurate optical reference system.

For qualitative evaluation, spatial distributions of the features in different environments were estimated. The relation to the objects along the propagation path and their influence on the feature outcomes were considered and interpreted.

For quantitative evaluation, the data were used to solve positioning tasks in the industrial environment using ML methods. The core idea of this approach is to formulate the position estimation as a supervised classification problem. The classification is simplified to a few target classes. This is possible because the positioning in the entire room is divided into individual partial areas (cells). Each cell then corresponds to a specific area in space, and each cell implements a separate classifier that again separates cells in that cell. Thus, the strength of simple quasi-binary classification of few data and two to a few classes can be exploited. Therefore, two different scenarios were used for the evaluation: First, the space for macroscopic positioning was divided into areas that can be categorized semantically. A supervised classification problem of these areas using the proposed features was formulated. The evaluations were made hierarchically with decreasing region size or with increasing position accuracy. Second, to evaluate the proposed features and demonstrate the applicability of the feature-based approach, CIR data were examined that represent the steps of a work process consisting of spatially separate screwing operations on a car door. The reliability and customization of the models were examined using additional datasets containing environmental changes. In all experiments, it could be shown that the hierarchical classification of extracted features from CIR signals of both LOS and NLOS signal propagation is possible even without absolute and synchronized time measurements to a position accuracy of 0.1 m${}^{2}$ in more than 90% of all cases. Even if the environment changes, very little information about the new environment is needed to adapt the hierarchical classifiers to the new situation.

The modeling approaches are presented in Section 3. Section 4 defines suitable features. A qualitative spatial analysis of the contributions for each feature in relation to the arrangement of the objects within the environment is presented in Section 5. The quantitative assessment using ML methods is discussed in Section 6. Finally, the evaluation of the approaches proposed in Section 6.2 and Section 6.3 are presented before the conclusions and outlook.

## 2. Literature Review

With the emergence of modern radio technology, local RF-based positioning is increasingly used in many applications, such as industry surveillance [1], sports tracking [2], and wildlife tracking [3]. Preliminary work applied a variety of technologies and estimation methods on a huge number of applications [4]. This section presents only a short overview.

For a broad variety of applications, positioning is based on signal propagation characteristics such as ToAs, time-differences-of-arrival (TDOAs), and round-trip-times (RTTs). Other systems employ angle-of-arrival (AoA) estimates or other angular measurements. More recently, to meet the propagation challenges provided by more chaotic environments, so-called fingerprinting approaches have emerged, which use a collected database of known observations, e.g., the received signal strength (RSS) of multiple anchors. This information is then used to train either a classification or regression algorithm in a supervised manner that uses the positions as labels and RSS data as features. Here, fingerprinting is an application of machine learning in radio-based positioning. Fingerprinting approaches have been proposed for different signal families, e.g., WiFi-fingerprinting [5] in challenging propagation scenarios at an accuracy of up to <1 m. Channel impulse response modeling is a common research topic in digital communications [6]. Here, modeling approaches, both statistical and deterministic, have also been proposed and validated [7]. The information that a CIR embeds has been used in various ways, e.g., the detection of scattering objects [8] or the adaption of tracking filters [9]. UWB channels in industrial environments have specifically been researched in [10]. Many other modeling approaches have been proposed for UWB signals in different environments (e.g., [11]). Impulse response analysis for audio signals also generated models, e.g., spatial audio reproduction models in multimedia applications [12].

In recent years, more and more machine- and deep-learning (DL) methods have been applied to positioning problems with various sensor signals [13,14,15]. Feature-based ML and DL approaches have both been used to identify LOS/NLOS and other propagation conditions for UWB positioning system [16,17,18,19,20,21]. CIRs have been used to estimate errors and signal quality and to enhance classic tracking techniques such as Bayesian filters [22,23,24]. To the best of our knowledge, all previous work use CIRs in combination with absolute time information to estimate a position with DL approaches. For example, the authors of [25,26,27] used convolutional neural networks (CNN) or other deep learning approaches and showed promising results, albeit with a very large amount of data and a complex network structure (at the cost of computational expense). A feature-based approach for discrete positioning, based on propagation models, has been proposed in [1]. This article is based on their work and also presents additional analysis and evaluation.

Most of the preliminary work either use “black-box” methods, when generalization is questionable or require absolute costly time information. Instead, we identify features that are in line with theoretic models for CIRs in adverse environments. Hence, we allow for a compact and comprehensive representation of the contained information. To achieve that, we analyze the characteristics of UWB CIRs in propagation environments with different degrees of complexity. Thus, we identify suitable features that enable positioning in industry environments with ML methods and analyze them with respect to their performance in different localization scenarios.

## 3. Modeling Channel Impulse Responses in Adverse Environments

In the following, the properties of CIRs are described in a simplified manner, for a more detailed explanation (cf. [6]), but phase information is not considered, as the used recording setup was only able to obtain the real parts of the CIRs.

Usually, CIRs are obtained by the transmission of a pseudo-random sequence s(t) known at both the transmitter and receiver. s(t) is chosen such that its autocorrelation in the time domain is close to a Dirac delta function:

(1) s(t)∗s(−t)≈δ(t).

This property can be exploited to obtain an estimate of the signal propagation channel. A simple model for the propagation of the signal is convolution of s(t) with the CIR, such that the received signal y(t) is given by:

(2) $$y\left(t\right)=s\left(t\right)*h\left(t\right)+n_{y}\left(t\right),$$

where ∗ denotes convolution. $n_{y}\left(t\right)$ represents the noise components not related to the propagation path, e.g., sensor noise and correlation artifacts, modeled as zero-mean white Gaussian noise for simplicity. An estimated CIR $\hat{h}\left(t\right)$ is obtained by the decorrelation of the received signal y(t) with the known sequence:

(3) $$\hat{h}\left(t\right)\approx y\left(t\right)*s\left(-t\right)+n_{h}\left(t\right).$$

In general, s(t) is chosen such that the amount of decorrelation artifacts is kept low. However, the combination of the artifacts with sensor noise leads to a level of spatially uncorrelated random noise $n_{h}\left(t\right)$ within the estimated impulse response. Apart from this, CIRs contain information on all the paths on which the signal traveled from a transmitter to a receiver, whether they are caused by reflection, diffraction, or scattering. Hence, a CIR contains information on both the propagation conditions and the position of the receiver and transmitter within the environment. However, the degree of the contained information can vary greatly: in a free-space case, theoretically, only one path is contained, while, in a complex setting, such as a typical industrial environment, many multipath components are included, leading to a more complex pattern. A simple analytical model for CIRs, in a reflective environment with $N_{p}$ signal paths, that is characterized by their signal propagation time $T_{i}$ and the signal attenuation $D_{n}$ along the path is given by:

(4) $$h\left(t\right)\approx \sum_{i=0}^{N_{p}}\delta \left(t-T_{i}\right)10^{\frac{D_{i}}{20}}.$$

The attenuation is defined as

(5) $$\begin{array}{c} D=20\log_{10}\left(\frac{4\pi df_{c}}{c}\right)+\sum_{i=0}^{N_{r}}R_{i}, \end{array}$$

containing both the free space path loss caused by the length of the path *d* and the absorption losses $R_{i}$ caused by the surfaces of each of the $N_{r}$ reflections. Of course, this model does not account for absorption, scattering, and diffraction, which are harder to model than reflections. An empirically inspired model taking more of these complex propagation effects into account is the expanded Saleh–Valenzuela model [28], which adds a tail of $N_{e}$ so-called clusters with exponentially decreasing magnitude, indexed *k* for each of the $N_{r}$ reflections with index *i*:

(6) $$h\left(t\right)\approx \sum_{i=0}^{N_{r}}\sum_{k=0}^{N_{e}}\delta \left(t-T_{i}-\tau_{i,k}\right)10^{\frac{D_{i}}{20}}e^{\tau_{i,k}/\gamma }.$$

The time-shifts of the primary reflections are given by $T_{i}$, while an additional time-shift $\tau_{i,k}$ is present for each of the cluster components. This model fits measured impulse responses in indoor environments based on the parameter γ and has been validated empirically.

## 4. Deduction of Relevant Features

**Figure 1:**
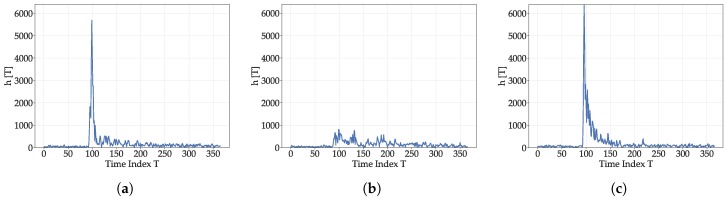
Magnitudes of exemplary CIRs. (**a**) LOS scenario. (**b**) NLOS scenario. (**c**) LOS scenario with multipath components.

While the simplified models introduced in Section 3 only cover a part of the actual propagation effects, they show that, apart of the ToA (which, usually, corresponds to the time of the first correlation peak above a certain threshold), a lot of additional spatial information related to the environment is contained in CIRs. While this information has been used by deep neural networks and using complete CIRs [26,27], this article proposes a more compact and comprehensible representation of the information by feature extraction. In the following, some approaches to represent this information are identified. In this section, the features are introduced and calculated for a set of three CIRs with different properties (LOS, NLOS, and multipath), as shown in Figure 1. The CIRs were collected with the measurement setup described in Section 5.1.1. The recording hardware setup did not allow for a representation of the CIRs in physical units, which also holds for the extracted features. This is not an issue for the proposed positioning approach, as it includes the normalization of the features. Hence, throughout the discussion of the evaluation and results, graphs my not explicitly denote physical units or units at all. The magnitudes of the obtained signals are presented. While the two CIRs with a LOS component (Figure 1a,c) exhibit a clearly identifiable peak (at *T* = 100), the CIR without a LOS connection (Figure 1b) does not. The LOS CIR with multipath components (Figure 1c) has a exponentially decreasing tail of early reflections at T∈[100;150] (similar to the model presented in Equation (6)) after the LOS peak, while, for the pure LOS CIR (Figure 1a), a much steeper decay is present. In the following, the used features are described, starting with simple basic ones (Correlation Maximum and Energy), followed by more sophisticated features related to energy decay (Decay Time Index and Peak Decay Exponential) and windowed features (time–frequency features and echo densities).

### 4.1. Basic Features

First, some basic features are introduced. Exemplary values are listed in Table 1. The energy $ENG\left(h\left(t\right)\right)=\int_{t}h\left(t\right)$ of the CIR is of interest, as it is affected by the path-loss, the presence of multipath components (MPCs), and absorption. The correlation outcome max(h(t)) was also chosen, as it varies greatly depending on location, especially between LOS and NLOS areas and is also related to the distance. For the CIRs in Figure 1, the maxima of the CIRs with LOS components at 5682 (Figure 1a) and 6362 (Figure 1c) are much higher than for the NLOS-CIR (Figure 1b) at 816. The energy also varies greatly: total values of about 69,700 for Figure 1a and about 82,300 for Figure 1c stand in contrast to only 54200 for Figure 1b. The reflections in Figure 1c introduce additional energy, while Figure 1b has no distinct high energy LOS peak.

### 4.2. Energy Decay

**Figure 2:**
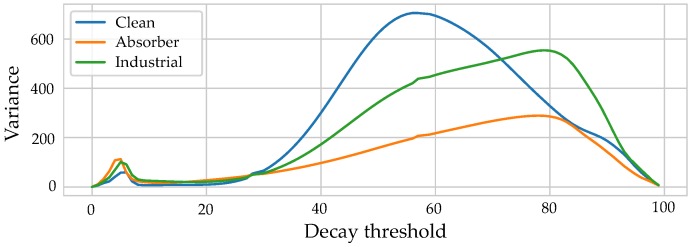
Variances of the energy decay values of the spatial distributions of the energy decay values corresponding to different percentiles.

The energy decay of an impulse response describes the cumulative integral of the contained energy over time, i.e., how much energy is still left in the impulse response after a certain amount of time. It is given by

(7) $$EDC\left(t\right)=\int_{t}^{t_{end}}h{\left(t\right)}^{2}dt,$$

where $t_{end}$ denotes the end of the impulse response. It has been studied in audio signal processing [29] and is usually used in connection to the reverberation time $T_{60}$ [30], a useful parameter for echo cancellation and other audio applications. For a compact representation of the EDC as a single feature, different characteristic values were extracted. Suitable percentiles were obtained by examining the statistical distribution of the EDC percentiles within a given dataset. The percentiles corresponding to the greatest variance in the distribution of the features were chosen by calculating the variance of the EDC in 1% steps. Figure 2 shows the resulting variances for the three datasets (Clean (pure LOS), Absorber (systematic reflections and absorptions), and Industrial (realistic environment)) that are described in detail in Section 5.1. A value of 78% resulted in the highest variances for the scenarios with more complex propagation patterns (i.e., a scenario with systematically introduced reflections and absorption and a realistic industrial environment). The peak for the clean scenario was not included, as, apart from the LOS components, no spatial information is present, such that the energy decay is mostly dominated by spatially uncorrelated noise.

For the CIRs in Figure 1, the percentile is reached at *T* = 210, 246, and 146 for the CIRs in Figure 1a–c. In the LOS CIR (Figure 1a), the contrast of high energy content of the LOS component to the rest of the CIR mostly containing correlation artifacts and noise causes a rapid energy decay in contrast to the two CIRs with strong multipath components.

### 4.3. Characteristic Exponential Function

In [28], empirical analysis of CIRs concludes that they are arranged in clusters of exponentially decreasing amplitudes as described in Equation (6). Based on this assumption, an exponential decay function can be defined occurring after the first peak in the CIR:

(8) $$CEF\left(t\right)=A_{p}\exp\left(-\sigma t\right),$$

where $A_{p}$ is the amplitude of the first peak (i.e., MAX) and σ defines the (unknown) steepness of the exponential function. Hence, σ can be estimated and used as a feature and will be called peak decay exponent (PDE) in the following. To obtain PDE, first, significant peaks in the magnitude of the CIR have to be identified. Then, using least-squares fitting, σ can be estimated. For more complex environments, σ should be smaller than for pure LOS environments. For the example CIRs, the estimated exponentials for the two CIRs with LOS components are −0.20 for Figure 1a and −0.0145 for Figure 1c, indicating a steeper decline for the pure LOS CIR. The value for the pure NLOS CIR is even lower at −0.011 as the peak is only slightly above the noise floor. While the number of parameters for obtaining σ (i.e., peak detection and fitting parameters) is quite high, it allows for a simple representation of the decay behavior CIR with one scalar value. This model could theoretically be expanded to multiple exponential functions as covered in Equation (6).

### 4.4. Echo Density Profile

**Figure 3:**
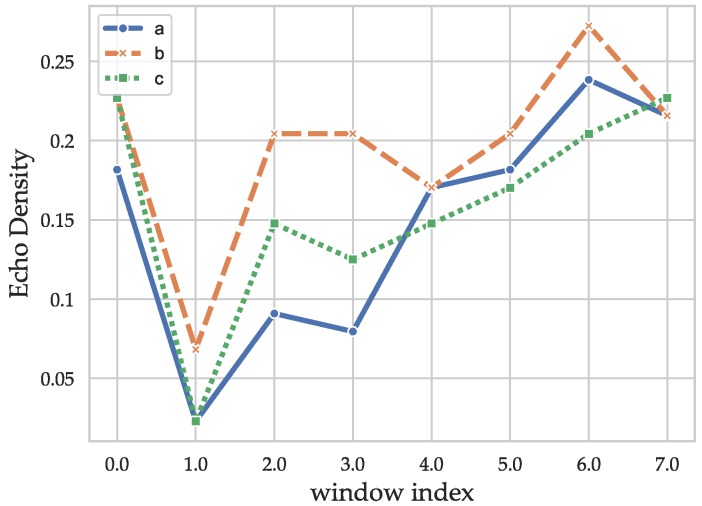
Echo densities of the exemplary CIRs (Figure 1).

The echo density profile has been introduced as a method to characterize different reverberant environments with respect to their geometry and the presence of scattering objects in audio signal processing [31]. It is given by the number of values outside the standard deviation within a sliding window w

(9) $$EDP\left(t\right)=A\sum_{\tau =t-\delta }^{t+\delta }w(|h\left(\tau \right)>\sigma_{\tau }\left|\right),$$

where *A* is a scaling parameter related to the statistic likelihood of an outlier that is not considered in the following as the features are scaled. $\sigma_{\tau }$ describes the standard deviation of the current window. In addition, a weighting function is applied. The echo density profile, similar to the energy decay is not a compact representation of the information contained in the CIRs and has to be compressed further. Thus, the windows were chosen with an overlay of only 50% to capture the difference in propagation behavior of the distinct phases while constituting a compact representation. The echo densities were calculated with a hamming window of size 128. To still keep the dimensionality low, all the echo densities were processed into one feature using a Principal Component Analysis (PCA). The echo densities of the example CIRs are shown in Figure 3. After a very low value caused by the variance increase due to the LOS component (leading to a lower amount of values above the standard deviation), an effect that is smaller for the NLOS CIR in Figure 1b, the values increase faster for the CIRs with MPCs (Figure 1b,c) and converges to a similar value for the end of the CIR, which mostly contains noise and correlation artifacts.

### 4.5. Time-Frequency Domain Features

Representing the CIRs in the frequency or time–frequency domain is also a promising way of extracting the contained information. The assumption is that the overlapping windows of the time-frequency domain representation contain information on the different phases of signal propagation contained in the CIR, which should reflect in the frequency domain. For example, if a signal has dominant early reflections, the tonality in the corresponding windows is higher. For this, the librosa library [32] was used for feature extraction. The features are calculated on the spectrogram of the CIRs, which is a time-frequency representation containing the squared magnitude of the short-time Fourier transform. This turns the signal into a set of frequency domain windows of specified length that each represents the spectral information within a short time interval. A Hann window of size of 128 with an overlap of 64 allowed for capturing the spectral information on the different characteristic phases in the impulse response. These settings present a trade-off between time-frequency resolution and compactness. The following features were evaluated (formal definitions of the spectral features are omitted for conciseness): the spectral centroid (SCD), corresponding to the center-of-mass of the spectral representation; and the spectral bandwidth (SBW), describing the frequency range of the majority of the signal. The spectral flatness (SFL) was also chosen, which is a measure of the atonality of the signal, i.e., the flatter is the spectrum, the more noise-like is the signal. Furthermore, the spectral roll-off (SRO) corresponds to the frequency below which the majority of the energy is contained.

**Figure 4:**
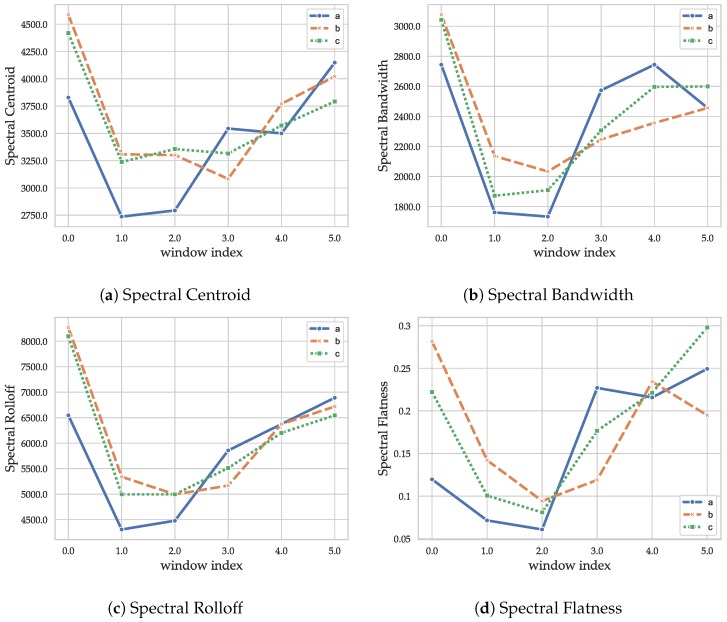
Outcomes of the spectral features.

The results of the time–frequency features for the CIRs in Figure 1 are shown in Figure 4: The spectral centroid (Figure 4a) of the pure LOS CIR (Figure 1a) is lower for window indices 0, 1, and 2. The CIR with both LOS and NLOS components (Figure 1c) differs from the pure NLOS CIR (Figure 1b) in window 3. The lower amount of early reflections explains the low outcome for the LOS CIR. The behavior in the latter half is similar for all the CIRs, as spatially uncorrelated noise dominates those late phases. The spectral bandwidth of windows 0, 1, and 2 is lower for the pure LOS CIR (Figure 1a), indicating that the behavior in these windows is less noise-like, which fits the model assumptions. The absence of earlier, spatially coherent reflections in windows 3 and 4 leads to a more noise-like outcome for the LOS CIR (Figure 1a), while the CIRs with multipath components (Figure 1b,c) still contain characteristic reflections that lead to a smaller spectral bandwidth as these have a more tonal and less noise-like behavior. The spectral roll-off exhibits a difference between LOS and NLOS in the early phases, but shows a low level of difference in the other phases, indicating that it is probably less suitable than the other features. However, no exhaustive search was conducted with respect to the roll-off percentage (which was set to 85%). The spectral flatness (Figure 4d) supports the hypothesis made for the outcomes of the spectral bandwidth, as it indicates that the pure LOS CIR (Figure 1a) transitions faster (in time window 3) into a noise-like phase due to the lack of early reflections. The large spread in the last time-window is significant; the NLOS CIR (Figure 1b) still contains some distinct albeit small peaks, which lead to more tonality in the signal. Overall, the time–frequency features add a different kind of information to the feature set than the other windowed feature, echo-density profile. In combination, the distinct behavior of the different phases (LOS-path, early reflections, and late reflections) should add information that is not contained in the basic and decay-related features. However, the information content of the spectral features is similar, so that using them directly would lead to an over-representation of the information they contain, as the dimensionality would be higher than for the basic and decay related features. To avoid this, it may be advantageous to apply dimensionality reduction techniques such as a PCA. However, with a measurement setup with the ability to collect CIRs at a higher rate, the resulting increase in possible frequency resolution may yield more informative time–frequency features. Furthermore, a thorough investigation of window types and sizes, FFT length, and overlap size could yield a more informative representation.

## 5. Spatial Analysis

To enable further analysis of the relation of the proposed features to the environment, in the following section, spatial distributions of the features in different environments are presented. Using these distributions, the relation of the proposed features to the environment and the positions of the tracked object and infrastructure tags can be evaluated in a qualitative manner.

### 5.1. Data Acquisition (Large-Scale Datasets)

**Figure 5:**
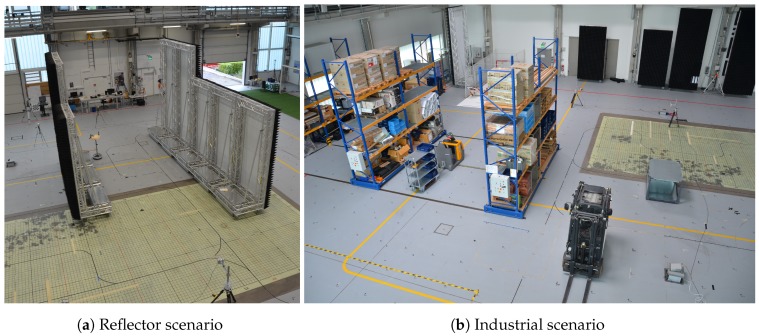
Real-world environments for the large-scale datasets.

**Figure 6:**
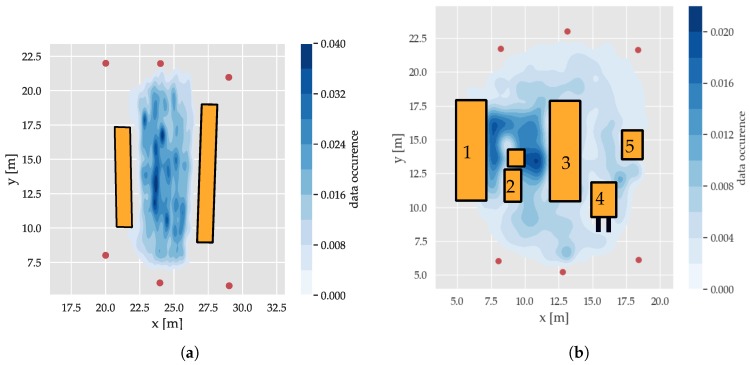
Probability distribution over the x and y positions of the datasets and positions of receivers (red) and relevant objects (orange). (**a**) Reflector scenario with the (r/a) walls (orange) see also Figure 5a. (**b**) Industrial scenario with the interfering objects (orange) see also Figure 5b.

For the spatial analysis of the features in relation to the environment, three datasets of about 50,000 CIRs per infrastructure tag (i.e., anchor or node) were recorded. First, a dataset in a free-space environment was collected in an area of about 5 m × 10 m. After that, in the next measurement, reflections and absorptions were systematically introduced using absorber/reflector (a/r) walls, leading to a reverberant environment with predictable multipath propagation, shown in Figure 5a. The final large-scale dataset represents a realistic industrial environment on an area of [14 m × 18 m], including large metal shelved filled with goods and industrial vehicles, as depicted in Figure 5b. The measurements were conducted by walking within the region with the transmitter for an extended period of time, as depicted in Figure 6. The distributions of data occurrence for the different datasets are shown in Figure 6: For the industrial scenario, the data collection focused on the area between the two storage shelves (objects (1) and (3) in Figure 6b) as this area had the most complex arrangement of interfering objects. In the case of the reflector dataset depicted in Figure 6b, data collection was focused on the area between the r/a walls and the transitions to the outer side to capture data with distinctive propagation channels.

#### 5.1.1. Hardware and Firmware Setup

For collecting the CIR data, a hardware module containing the decawave chip DW1000 using impulse radio UWB technology with adjustable center frequencies between 3.5 and 6.5 GHz and bandwidths between 0.5 and 1.3 GHz was employed. The chips can be configured as transmitters and receivers, dependent on the installed firmware. In total, eight tags were available of which 6/7 were used as infrastructure tags and one as a mobile tag. The mobile tag was configured as a receiver for the dataset used in Section 6.3 and as a transmitter for the other evaluations (the opposite holds for the infrastructure nodes). For the large-scale datasets used in Section 5 and Section 6.2, the Nikon iGPS system (with a positioning accuracy in the range of millimeters) was used as a reference system and synchronization of the CIRs and reference data was done by transmitting the data to a global server over a TCP/IP protocol.

### 5.2. Method

**Figure 7:**
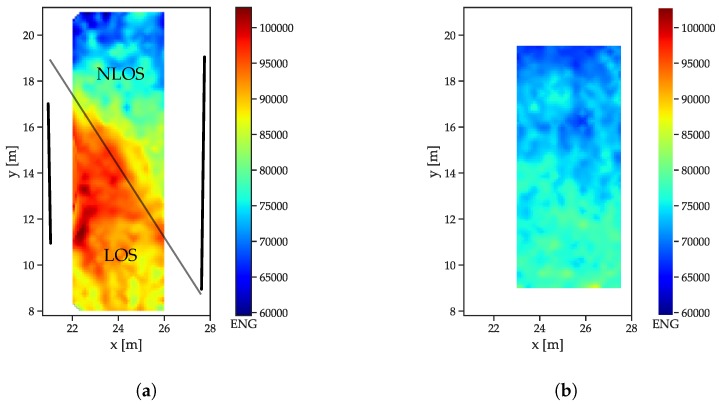
Energy index distributions. (**a**) Reflector scenario. The reflector walls are indicated by the thick black lines, the thin black line marks the transition between LOS and NLOS. (**b**) Free space scenario.

**Figure 8:**
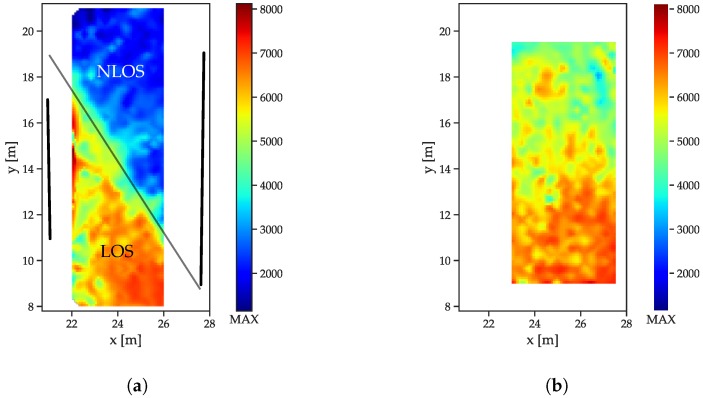
Correlation maximum distributions. (**a**) Reflector scenario. The reflector walls are indicated by the thick black lines, the thin black line marks the transition between LOS and NLOS. (**b**) Free space scenario.

**Figure 9:**
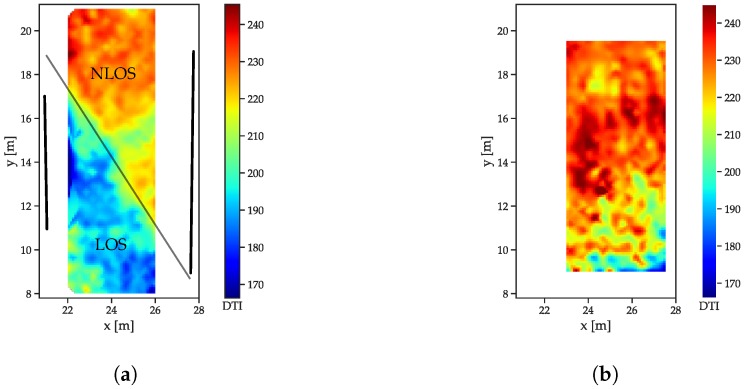
Decay time index distributions. (**a**) Reflector scenario. The reflector walls are indicated by the thick black lines, the thin black line marks the transition between LOS and NLOS. (**b**) Free space scenario.

**Figure 10:**
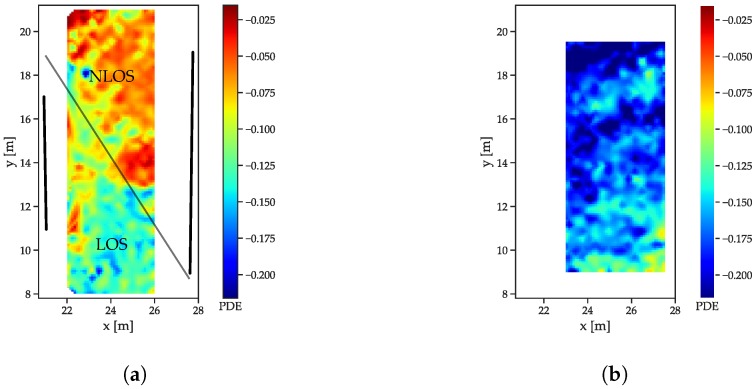
Decay exponent distributions. (**a**) Reflector scenario. The reflector walls are indicated by the thick black lines, the thin black line marks the transition between LOS and NLOS. (**b**) Free space scenario.

**Figure 11:**
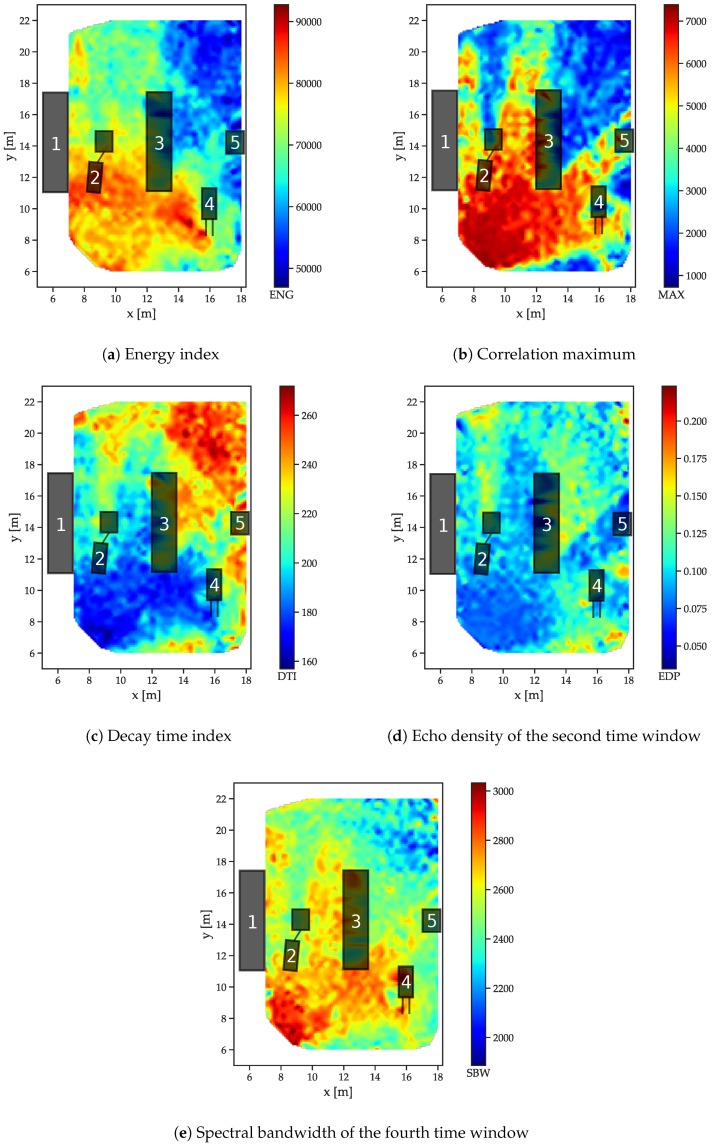
Feature distributions for the industrial dataset.

For qualitative assessment of the usability of the features for positioning, first, some spatial distributions over the large scale datasets are presented. To obtain the spatial distributions, the data were separated into a grid of tiles of size [30 cm × 30 cm]. The means of the feature outcomes within the grids were then used for estimating a distribution over the whole area using piece-wise cubic interpolation. For conciseness, only some exemplary distributions are shown in Figure 7, Figure 8, Figure 9, Figure 10 and Figure 11. The complete set will be made available in the Supplementary Materials.

### 5.3. Influence of LOS/NLOS on the Features

To compare the behavior of LOS/NLOS CIRs, two large scale datasets (see Section 5.1) were used: the free-space scenario and the dataset with the reflector/absorber (r/a) walls: The arrangement of the absorber walls was chosen such that, for each of the infrastructure tags, parts of the LOS signals was absorbed and CIRs both with and without MPCs were available. The r/a walls, as shown in Figure 5a, have a metal plate on the reflector side, with some metal mechanical arrangements towards the bottom side that cause some perturbation. Both scenarios were recorded with the same receiver setup, but in a slightly different area, such that, for the free space scenario, enough data for interpolation were only available in an area shifted 1 m in the positive x-direction. All feature distributions are shown for a receiver located at (29 m, 5.8 m). The energy distributions (Figure 7a) show that the LOS path yields a much higher amount of energy than the NLOS components (Figure 7a), as indicated by the drop-off in energy. The fact that separation between the LOS and NLOS areas is not a straight line is due to diffraction at the edges of the reflector/absorber walls (marked as thick black lines). Furthermore, the reflections, especially at the left absorber wall, cause a much higher energy content in the CIR, especially close to the wall and where the reflections are present in addition to the LOS component. The free space CIRs exhibit a much more homogeneous energy distribution (Figure 7b), with the energy, slowly decreasing with the distance to the receiver (and therefore with the size of the correlation peak that can be assumed to be the main contributor to the overall energy).

The correlation maxima (Figure 8) exhibit an even more distinguishable difference between LOS and NLOS CIRs: while the outcomes of the LOS CIRs decrease radially with the distance, a sharp distinction occurs between the areas affected and unaffected by the absorber wall (Figure 8a).

The decay time indices (Figure 9) increase with the distance in the free space scenario (Figure 9b). The decreasing power of the LOS peak results in a decreasing contribution to the total energy. The r/a walls cause a slower decay in the NLOS cases (Figure 9a), as the contribution of the LOS peak is not present.

For the peak decay exponential (Figure 10), the much lower exponents indicate the faster decay for the LOS case (Figure 10b) compared to the NLOS areas in Figure 10a. The much slower decay for the region without a LOS connection also is in compliance with the models presented in Section 3.

### 5.4. Influence of Scattering and Blocking Objects in the Industrial Scenario

The spatial analysis of the data obtained in the industrial scenario is not as straightforward as for the free-space and reflector scenarios, as regions with distinct propagation patterns are not easily identified from the measurement setup due to the complex shapes and variety of materials. Therefore, the goal is to find out how strong the relationship between the feature outcomes and the environment is by examining the spatial distributions in relation to the arrangement of influencing objects. The examples are shown in Figure 11 for a receiver located at (20 m, 8 m). The energy of the signal shows a distinct relation to the environment (see Figure 11a): behind shelf (3), absorption by the objects within leads to a high drop in energy.

The effect of the missing LOS is even higher for the correlation maxima (Figure 11b) so that even the small vehicle (2) has a distinct “shadow” caused by the lack of a direct signal path with blocking objects in the LOS. The decay time index Figure 11c also increases in areas with objects causing multipath propagation, such as between metal shelfs (1) and (3) and, especially behind metal shelf (3), where it can be assumed that no or only a strongly attenuated LOS signal component is available.

For the windowed features, the second time window of the echo densities (which typically includes the LOS peak if it is present in the data) in Figure 11d also shows some distinction between LOS and NLOS, while no clear relation to the distance between transmitter and receiver is present. In the fourth time window, the spectral bandwidth also shows a clear distinction to the arrangement of objects in the environment and their geometric relation to the location of the receiver.

### 5.5. Discussion

In conclusion, there exist distinct relations between the feature distributions and the environment. Areas with both characteristic absorption and reflection patterns exist and these differences are project onto the features. The clear relation of the signals to the environment indicates that a localization approach based on the proposed features may actually perform better in more complex environments, as objects with influence on the propagation actually create more diversity in the spatial distributions, especially if they are available from multiple infrastructure tags. Because of these anomalies, the spatial distributions are much more inhomogeneous than the spatial distributions for a typical free-space distance-related measurement (which just increases radially from the receiver). Unlike pure anomaly-based methods such as magnetic field based Simultaneous Localization and Mapping (SLAM) [33], the feature distributions also contain information on the infrastructure tags. Hence, infrastructure can be installed with the specific purpose of creating spatially diverse distributions. The clear relation to the environment indicates that it should be possible to generate a map of the environment using the feature-based approach, especially when combined with additional information sources, such as semantic maps [34] or object information detected by other sensors such as cameras [35]. Furthermore, the impact of the propagation conditions, as described in Section 5.3, and the spatial behavior of the features implies that the proposed features are usable to classify propagation conditions (MPC, LOS, and NLOS detection), and enhance the performance of tracking filters [18]. The spatial distributions of the features, while informative and spatially significant, exhibit a degree of relatedness that implies that a more compact representation can be obtained. Thus, further studies in dimensionality reduction using classical methods, such as PCA, or neural network architectures, such as variational autoencoders [36], may allow for a more compact representation of the spatial information.

After the behavior of the features in different environments was qualitatively evaluated, a quantitative evaluation of their suitability for positioning was conducted.

## 6. Evaluation

After the qualitative assessment of the proposed feature-based approach in Section 5, the features were applied to positioning scenarios. After pre-processing the data, the features were extracted and post-processed for the ML algorithms. The features were then grouped to allow for a separate assessment of and the distinction between different kinds. The first evaluation was conducted using the “Industrial” dataset (see Section 5.1). The dataset was separated into regions of decreasing size that were set up for hierarchical ML classification. After that, in the second evaluation, the distinction between a set of the positions of screw processes on a car was considered. A separate dataset was introduced, including different changes in the environment. Training was conducted with both the original dataset and a mix of the original dataset and the datasets including the environment changes. Finally, a conclusive assessment of the introduced features was conducted. In terms of the application for industrial surveillance, the region identification can be interpreted as the detection of the object that a worker is working on (i.e., a macroscopic positioning) and the work process localization is then related to the various work tasks that are conducted on the object.

### 6.1. Evaluation Framework

**Figure 12:**
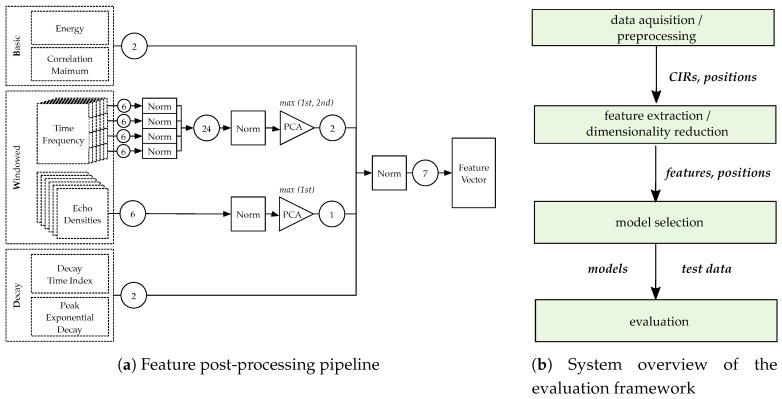
Overview of the applied ML pipeline.

The evaluation setup is depicted in Figure 12b: First, the collected data were pre-processed. Samples that did not contain CIRs from all receivers/transmitters were discarded as they could not provide complete feature vectors (amounting to about 20% of the data). Then, the recorded data, containing CIRs from all infrastructure tags, reference positions, and timestamps, were aligned such that a matrix of CIRs ${\left(h_{1,i},\cdots ,h_{N_{r},i}\right)}^{T}$, and corresponding reference positions $x_{i}$ was available. Afterwards, this set of labeled data was then processed into the feature extraction and post-processing framework. The features were prepared for use in solving a classification problem. We split the set of proposed features sets into three different categories: *B*asic, *W*indowed, and *D*ecay. *B* contains a single energy index (ENG) and a single correlation maximum (MAX) feature. *W* compromises both the time–frequency (SCD, SBW, SFL, and SRO) and echo density (ED) features. There were 24 time–frequency features, four different groups of six features each, and six ED features. *D* includes two features: the Decay Time Index (DTI), and the Peak Decay Exponent (PDE). The process for creating the final feature domain representation is shown in Figure 12a. First, all the time–frequency domain features were normalized separately as the value ranges of their outcomes varied greatly. Then, the features were normalized together. The six ED features were also normalized.

To reduce the dimensionality, i.e., to compress the feature space to a lower number of features while keeping significant information and characteristics of the features, we determined the principal components of both the 24 normalized time–frequency features and the six normalized echo density features. We analyzed the principal components (PCs) and selected the maximal component of the *echo density* features and the features corresponding to the first and second PCs of the time–frequency features. The resulting feature-space representation consists of seven features, two *B*, two *W* time–frequency, one *W* echo density, and two *D* features. Again, we normalized these seven features to obtain the final feature space representation. This process was done for each of the infrastructure tags, leading to 34 features/tag before and 7 features/tag after the PCA. For the six infrastructure tags used in the measurements, the resulting dimensionality was $N_{feat}\times N_{anch}=7\times 6$. After that, the evaluation models were selected: classifiers were trained and optimized with cross-validation techniques after separating the data into training, testing and validation datasets. The exact methodology and results of the evaluation methods are presented in detail in the following subsections.

### 6.2. Region Identification

For industrial surveillance, discrete or semantically motivated positioning is of importance because tasks are typically confined to certain areas. Therefore, for the evaluation, the focus lies on the classification of regions. We exploit the fact, that a single classification model suffers from rising uncertainties with an increasing number of possible classes. Therefore, we reduce the number of classes (note, in the following, a class represents a region), to reduce the possibility of uncertainty, but at the cost of several independent classification models that are hierarchically and semantically selected for each region. Thus, if a model classifies incorrectly, we also select an incorrect model to classify subregions. This hierarchical classification approach provides localization that allows for a semantic rather than a geometric interpretation.

#### 6.2.1. Hierarchical Machine Learning Pipeline

**Figure 13:**
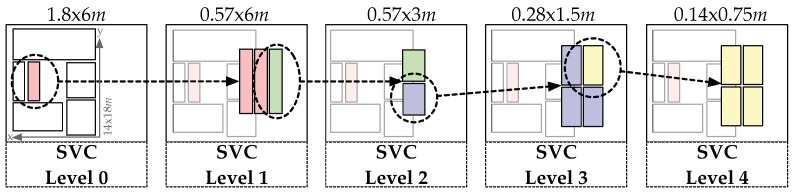
Hierarchical classes used in the machine learning pipeline. Number of classes per region from the left to the right: 6 at *Level 0*, 3 at *Level 1*, 2 at *Level 2*, 4 at *Level 3*, and again 4 at *Level 4*.

On the highest level, the data from the industrial dataset are split into six regions (see Figure 13). The regions are selected such that they imply real-world relevance (i.e., a typical transition between storage shelves), but also include enough and equally distributed data points for the machine learning algorithms. The red box then shows the subregion that is further partitioned in *Level 1*.

Thus, if the model correctly classifies that the obtained signal originates from inside the red box in *Level 0*, another model that classifies the regions at *Level 1* can be applied. This process can then be repeated until *Level 4* is reached, which represents an accuracy of 14 × 75 cm, about the size of a typical work piece (localization of production steps on a work piece see Section 6.3). In Figure 6b, the distribution of collected data is shown. The area in between the storage shelves was selected, as it exhibited the highest data density and a high structural complexity. However, at *Level 4*, only about 100 data points per class were available.

#### 6.2.2. Machine Learning Models

In a preliminary, study, two classifiers were studied and optimized using grid-search methods: a Decision Tree Classifier (DT) and a Support Vector Classifier (SVC).

First, for the SVC model, preliminary experiments showed that, with a radial basis function kernel *k*, the parameters *C*ost = 19 and Γ = 5, and a polynomial order *p* = 5, SVC provides the highest accuracy and the most reliable results. Second, for the DT model [37], preliminary experiments showed that, for the ω-classification, a DT performs best when configured with the Gini diversity index *c* = $I_{G}$ as split criterion, a maximum depth of the tree of no more than $depth_{max}$ = 100, the minimal number of samples required to be at a leaf node $leaf_{min}$ = 1, and the minimum number of samples required to split a node $split_{min}$ >10.

For further evaluation, we selected the SVC as it yielded the highest accuracy on a 30%/70% (test/training) split of the data. However, we performed an intense hyperparameter optimization on both SVC and DT to find parameters that enable an optimal model for each *Level* or for all *Levels* (Hyperparameter optimization: SVC: *k* = rbf∈{linear,polynomial,gaussian,rbf}, *C*ost = 19 $\in \{10^{-5}:1:10^{5}\}$, Γ = 5 $\in \{10^{-5}:1:10^{5}\}$, *p* = 5 ∈{1:1:10}; DT: *c* = ${}^{\prime }gini^{\prime }$$\in {\{}^{\prime }gini^{\prime }{,}^{\prime }entropy^{\prime }\}$, $depth_{max}$ = 100 ∈{1:10:1000}, $leaf_{min}$ = 1 ∈{1:1:15}, $split_{min}$ ≥ 10 ∈{1:1:100}.).

#### 6.2.3. Results

First, we describe the optimization parameters for the model that operates on all levels and the models that operate individually per level. Then, we describe the results for both a cross-fold validation and a hold-out test. First, a single model was optimized for all levels (“one-4-all”), leading to a kernel coefficient Γ of 5 and a penalty parameter *C* of 19. In a second evaluation step, individual models were trained for each *Level* (“one-4-each”)(*Level 0* *C* = 7, Γ = 18; *Level 1* *C* = 17, Γ = 6; *Level 2* *C* = 5, Γ = 13; *Level 3* *C* = 21, Γ = 6; *Level 4* *C* = 18, Γ = 5.). The validation revealed that the data are very well embedded in each model, as shown in Table 2: a cross-fold of 10 showed accuracies over 90% for all levels. When training separate models per level, the model yields slightly higher accuracy of up to 99.8% at *Level 0*. To evaluate the generalization of the proposed model, the dataset was split for each level individually. Note, as the number of data points decreases significantly at higher levels, the datasets cannot be split equally for each level due to a lack of data. The applied splits (training/test) were 70%/30% for *Level 0*, 80%/20% for *Level 1*, 85%/15% for *Level 2*, 90%/10% for *Level 3* and 95%/5% for *Level 4*. The test revealed that the “one-4-all” model really struggles on unknown data. The best model showed an accuracy of 63.3% for *Level 0*. When training separate models per *level*, the model yields much higher accuracies of up to 98.7%.

The impact of the different features sets (as introduced in Section 6.1) on the accuracy of our models was also investigated. We trained different models on the following combinations of feature sets: *B*, *W*, *D*, (B,W), (B,D), (B,W,D), and (W,D). The hyperparameters were optimized for each of the combinations. Table 2 shows the accuracies of the model that performed best on all *Levels* and all feature combinations, while Table 3 includes the results with unknown data. If only the *W* features are used, the model performs worst (accuracy of 34% on *Level 0* to 49% on *Level 4*). Instead, by using only *B* or *D*, the model performs much better (67% on *Level 3* to 97% on *Level 2*). However, the model performs best if we combine *B* and *D* (up to 97%). Interestingly, although the *W* features do not add a significant contribution, they do contribute to a higher accuracy on *Level 4*.

#### 6.2.4. Discussion

The proposed models are able to accurately distinguish between regions if individual models are trained for all levels (“one-4-each”). The analysis of the impact of different features revealed that *B* and *D* seem to contribute most to a models accuracy. The energies and correlation maxima vary greatly as the interaction with the environment causes significant differences in the outcomes; the same holds for the decay-related behavior for the CIRs. Especially between larger regions, the differences in path loss, which are present in the *B* features, are also relevant. However, within smaller regions, it may be possible that *W* features really represent different signal characteristics, thus the model may really benefit from these features. This is investigated further in Section 6.3. The method is directly applicable for industrial positioning with manageable datasets and without the need for a reference system for the training procedure. In the following, the localization of production tasks in an area corresponding roughly to *Level 4* is presented.

### 6.3. Work Process Localization

**Figure 14:**
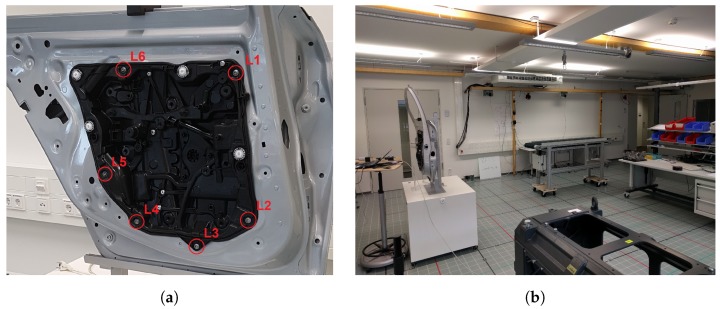
Pictures of the test environment for the small-scale datasets. (**a**) The location of the positions-of-interest (L1–L6) on a car door. (**b**) The environment for the small-scale dataset.

In industrial applications, the position and order of work processes is especially relevant for quality assurance [38]. This means that the semantic information implied by the positions (i.e., the work process) is of interest. Furthermore, an industrial environment will usually change over time. Hence, the performance a ML or fingerprinting approach can deteriorate heavily [1]. To employ a running data driven positioning solution in this kind of environment, the models are retrained to adapt changes in the environment. Therefore, a classification task was setup for the distinction between a discrete set of work processes (i.e., screwing the screws into positions on a car door shown in Figure 14a). The classification process and evaluation setup follow the structure introduced in Figure 12b. First, CIRs from the infrastructure tags are recorded. Then, the features are extracted from the signals. After splitting the data into training, test, and validation sets, evaluation was conducted for the different scenarios described later in this section. Classical machine learning models were chosen for the evaluation: Support vector classifier (SVC) with s radial basis function or linear kernels, a decision tree (DT) classifier and a random forest (RF) classifier [37]. A grid-search algorithm fount suitable classifiers and hyperparameters for the different evaluations. (Hyperparameter optimization: SVC: Kernel ∈{linear,rbf}, $C\in \{10^{-4},10^{3}\}$, Γ = $\in 10^{-5},10^{-4},...10^{0}$; DT: min.samples/split∈{2,3,...,15}, min.samples/leaf∈{1,2,...,11}; RF: $N_{est}\in \left\{10,15,...,30\right\}$, min.samples/leaf∈{1,2,3}, min.samples/split∈{3,4,...,7}).

#### 6.3.1. Data Acquisition

To demonstrate that the proposed features are suited for tasks in process monitoring, a dataset was recorded that captures the tasks of screwing six screws into a car door and considered changes in the environment. The locations of the screws on the car door are shown in Figure 14a and are spaced 15–36 cm apart.

**Figure 15:**
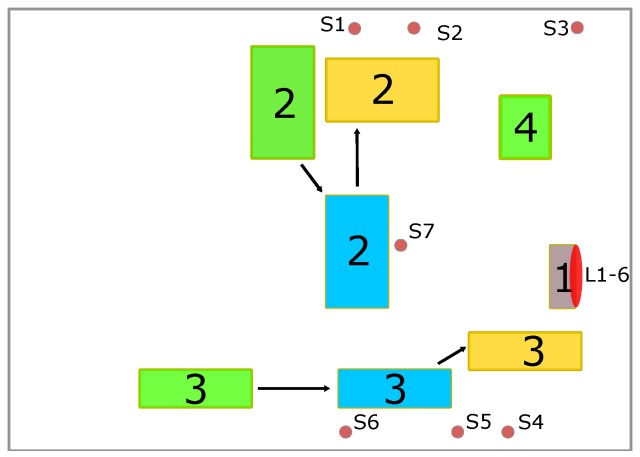
Layout of the evaluation. The positions-of-interest are placed on the car door (1), other relevant objects are a workbench (2), assembly line (3), and motor mounting bracket (4). Changes in the environment are displayed in different colors: Green shows the initial setup, while blue and yellow show the changes in the environment.

The proximity of the positions and the reflective behavior of the door make this a challenging task for classical positioning methods. The whole evaluation setup is shown in Figure 14b and Figure 15: the car door (1) is placed close to a wall in a room with the size of 14 m × 8 m × 2.7 m, with the screw positions facing the wall, leading to the absence of a LOS transmission path for most of the transmitters. Additionally, three objects are present causing reflections and absorption of the UWB-signal: a movable workbench containing an assembly setup and various tools (2), a mobile assembly line (3), and a metallic motor mounting bracket (4). The different colors indicate the different positions of the objects used in the different evaluation steps. The transmitters are mostly placed near the ceiling of the room; only transmitters S2 and S4 are placed near the floor. To evaluate the approach, several datasets were recorded after applying different changes to the environment. The data were recorded for each screw location. While recording the data, the receiver was moved and rotated slightly to emulate realistic conditions for screw tightening. For the first dataset (called “Ideal” in the following), the relevant objects in the environment are indicated by the green markers in Figure 15. Data were recorded for 6 min per screw location resulting in about 720 CIRs per transmitter. The dataset was then split into 30% test, 20% evaluation, and 50% training data.

In the next step, different changes were introduced to emulate an industrial scenario with more realistic propagation conditions. Objects were moved to different positions, shown in Figure 15 in blue (“Ch. Env 1”) and yellow (“Ch. Env.2 “), to change the propagation conditions. The first change was chosen to only alter the propagation conditions slightly, while the second change was more drastic, as two of the objects were arranged to block the LOS completely. In addition, an evaluation set (“Persons”) was recorded with the environment arrangement from the “Ideal” setting, but with group of five people walking within the environment, causing absorption. For each of the environment changes, 2 min of data were recorded per screw location, resulting in about 240 CIRs each. The datasets were then split into 25% training, 25% validation, and 50% test data.

#### 6.3.2. Evaluation

**Figure 16:**
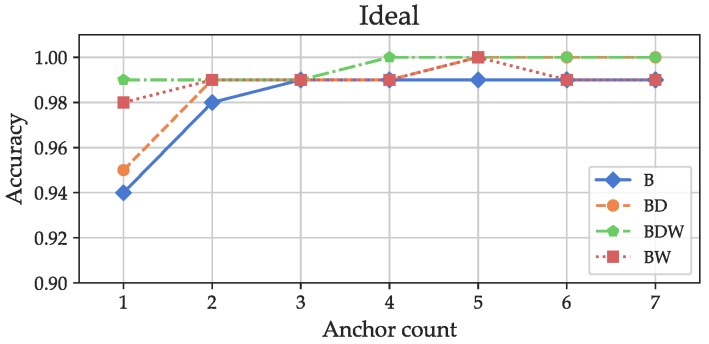
Classification accuracies for different anchor counts with the “Ideal” dataset for different combinations of features.

The two main objectives of the evaluation were to test the robustness and suitability of the feature based approach and to compare the features. Another objective was to find out how many and which receivers are needed for accurate discrete localization (a more detailed analysis of this aspect is contained in [1]). To achieve this, different evaluation settings were chosen: the features were grouped into sets (see Section 6.1). First, a model was fitted onto the training and validated with the validation dataset of the “Ideal” scenario. The classification results are shown in Figure 16: even for only one anchor, using all features (labeled BDW), an accuracy of about 99% was reached. The decay-related features (labeled “BD”) only yielded a slight increase in accuracy in comparison to only using the basic features (“B”) for one anchor. An accuracy of almost 100% was reached with an anchor count of 4 with all features. Evaluation of the “Ideal” datasets proved the validity of the feature-based approach: even with an infrastructure that would not be sufficient for positioning (a classical trilaterion based system needs at least three anchors to obtain a non-ambiguous result even with perfect propagation conditions), an almost perfect positioning accuracy can be reached.

**Figure 17:**
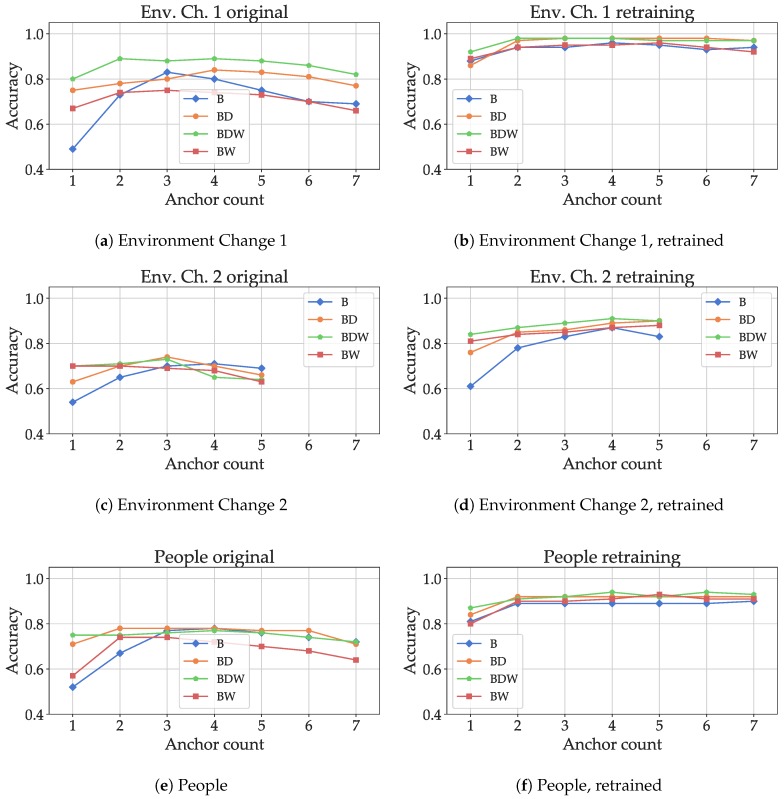
Classification accuracies for different anchor counts for testing with the datasets including environment changes. The results in the left column are without, in the right column with retraining.

In the next evaluation step, the model fitted on the “Ideal” dataset was then evaluated on test data from the “Other” datasets including environmental changes (“Persons”, and “Ch. Env 1”, “Ch. Env.2”). The results are shown in Figure 17a,c,e: for the small changes in the environment, using all features (B,D,W), an accuracy of 90% can still be reached with only two anchors. For all feature combinations, the classification accuracy decreases with higher numbers of anchors, as the smaller numbers only include the anchors for which the propagation path was only altered slightly. For environment change 2 (Figure 17c), only a maximum number of five anchors was available, as the propagation paths for the remaining two were blocked and not enough data were available for evaluation. The drastic change in propagation conditions (the bodies of five people causing absorption) led to a severe drop in classification accuracy, about 72% with three anchors and the combination of basic and decay-related features. In the final realistic scenario, the absorption caused by people walking in the propagation path resulted in decrease in performance, but results of about 80% accuracy could still be reached even for two anchors. In general, except for “Env. Ch. 2” with five anchors, additional features apart from the basic ones showed a significant increase in performance, especially for fewer anchors. The inclusion of both the windowed and decay-related features seems feasible. However, including only one of the two options outperformed using both in some cases, notably for anchor numbers above 2 with the “People” dataset. This is a similar observation to what was found out for the macroscopic case (see Section 6.2).

In the final evaluation step, data from the environment change scenarios were used to retrain the model: the training and validation data from the “Other” datasets were mixed with the “Ideal” one to generate new models, which were then evaluated against the test data from the “Other” datasets. The goal was to find out if the models can adapt to environment changes by using a low amount of data. For the small environment change (Figure 17b), the model adapts and results close to 100% were achieved. The feature set with only the decay-related features performed similarly to the full feature set. For the second environment change (Figure 17d), the full feature set produced the best results. Retraining increased the performance significantly, especially for higher numbers of anchors (for which the performance decreased for the model without retraining in Figure 17c), indicating that the model could adapt to the changes in the environment. For the “People” scenario (Figure 17f), retraining also resulted in an increase in accuracy; the maximum performance was already reached with two anchors. The high performance gain produced by retraining is probably also due to the fact that data from the environment changes were used as validation data for model optimization. Therefore, the share of the original data that was not or only slightly affected by the changes was weighted higher and still able to contribute positively. While the combination of all features produced the best results with a low number of infrastructure tags, both the windowed and decay-related features could individually improve the performance of the classification, with the exception of “Env. Ch. 2” and a high number of anchors. In many cases, the “W” features seemed to be necessary to achieve the maximal performance, especially for lower numbers of infrastructure tags, but the “D”-features also contributed significantly.

## 7. Conclusions

This article presents an analysis of the positioning features of ultra-wideband (UWB) channel impulse responses (CIRs). The goal is to find a suitable method for positioning within a discrete set of semantic regions, especially in regions with complex propagation conditions, such as industrial environments. First, some theoretical models of CIRs are introduced. Based on the models, different features with varying degrees of complexity are proposed. To analyze the features, different datasets were recorded using an optical reference system and a recording architecture based on the popular decawave chip: a dataset over an area of [5 m × 10 m] without any blocking or reflecting objects; an area of [5 m × 10 m] with an arrangement of absorber/reflector walls; and an area of [14 m × 18 m] in a realistic industrial environment featuring a variety of propagation-affecting objects. For qualitative spatial analysis, spatial distributions of the features were estimated using piece-wise cubic interpolation. While only some feature distributions exhibited a clear relation to the distance between transmitter and receiver, all of them showed a clear relation to the environment, in both the separation of LOS and NLOS behavior in the reflector/absorber dataset, and the influence of objects such as metal shelves in the industrial dataset. This leads to the conclusion that, unlike classical positioning systems, the proposed feature-based approach is especially beneficial in areas with higher structural complexity. A macroscopic positioning task for the separation of regions was conducted with the industrial dataset and a hierarchical structure of models, corresponding to increasing positioning accuracy. With dedicated classifiers, high accuracies of above 95% were achieved. Finally, the distinction between a small set of semantic regions (related to the work processes involved in the assembly of a car door) was evaluated. The dataset was collected in a realistic industrial scenario and different environment changes were considered. Different classification models using the proposed feature representation were then optimized to the original dataset. While a high accuracy of almost 100% was achieved on test data from the original dataset, the results deteriorated heavily on the test data of the other datasets. To adapt to environmental change, small amounts of training data from the other datasets retrained the models, leading to a steep increase in accuracy up to 98%. In terms of assessment of the proposed features, the sets of windowed features (containing dimensionality reduced echo densities and time–frequency domain features) had the smallest contribution to the overall results in the macroscopic evaluation, while the basic (energy and correlation maximum) and decay related features contributed significantly. However, the windowed features contributed positively to the accuracy of the work process localization task. The proposed feature based approach allows for a compact representation of the CIRs. It is independent of the positions of the infrastructure tags and therefore does not require a time-consuming and complex setup procedure. Furthermore, the data can be labeled within semantic regions rather than with actual positions. Thus, the approach does not require a positioning reference system and enables the models to quickly be adapted to environmental change.

## 8. Outlook

The proposed feature-based approach for positioning with UWB CIRs can be expanded by adding additional features or applying other dimensionality reduction techniques. Measurements of the phase information may yield additional information sources that have not been covered in this contribution. Other features may be better for representing the influence of interfering objects on the phase information. The proposed time–frequency representation could be improved by a thorough investigation of the various related parameters (e.g., window type/size, overlap size, and FFT length). Spatial distributions of the CIRs were used for visualization, but a representation based on Gaussian process regression [39,40,41] may directly yield a statistical model usable for positioning [42]. In the future, a comparison or combination of the proposed features with a deep learning approach will also be investigated, such as a comparison with the latent layers in a variational autoencoder [36]. A description of the spatial distributions using methods of quantifying the information, such as the Kullback–Leibler Divergence [43] or the Wasserstein Metric [44], could also yield a more comprehensible assessment of the features. Finally, a combination of the approaches with a dynamic model into a tracking filter or even a simultaneous localization and mapping (SLAM) [45] or semi-supervised learning [46] approach could yield a robust and accurate positioning solution. The evaluation of the proposed approach in a real industrial environment with a variety of tasks implying a semantic map [47] is also of interest.

## Figures and Tables

**Table 1:** Feature outcomes (Basic and Decay-related) for the example CIRs introduced in Figure 1.

Feature	LOS CIR (a)	NLOS CIR (b)	MP CIR (c)
Energy Index (ENG)	69,700	54,200	82,300
Correlation Maximum (MAX)	5682	816	6362
Decay Time Index (DTI)	210	246	146
Peak Decay Exponent (PDE)	−0.20	−0.011	−0.0145

**Table 2:** Accuracy of “one-4-all *Levels*” and “one-4-each *Level*” models. Highest values are bold.

Evaluation Design	One-4-All [%]					One-4-Each [%]				
	*L 0*	*L 1*	*L 2*	*L 3*	*L 4*	*L 0*	*L 1*	*L 2*	*L 3*	*L 4*
Cross-Validation	**98.2**	**96.3**	**92.3**	**95.4**	**91.2**	**99.8**	**98.3**	**99.6**	**94.9**	**97.6**
Hold-Out	63.3	56.1	74.3	48.2	53.1	91.2	71.4	98.7	73.1	81.4

**Table 3:** Accuracy per *Level* and feature set. “One-4-each” models on unknown test data. Highest values are bold.

Feature Set	Accuracy per *Level* [%]				
	*L 0*	*L 1*	*L 2*	*L 3*	*L 4*
*B*	87.1	69.9	96.6	**73.6**	89.47
*D*	80.9	59.7	89.8	72.7	73.6
*W*	51.2	43.1	69.1	46.36	52.6
B,D	**91.0**	*69.9*	**97.8**	71.8	**89.5**
B,W	85.3	61.9	96.6	72.7	73.7
D,W	79.8	54.5	84.8	60.9	68.4
B,D,W	90.9	67.9	97.7	70.9	78.9

## Supplementary Materials

The following are available online at https://www.mdpi.com/1424-8220/19/24/5547/s1.
